# Supplementary material for: Exploring the Thermostability of CRISPR–Cas12b using Molecular Dynamics Simulations
Source: ArXiv. 2024 Aug 20:arXiv:2408.11149v1. Preprint. [Version 1] (PMC11383325)
Supplement: Supplement 1 [file NIHPP2408.11149v1-supplement-1.pdf]

Supporting Information for

Exploring the Thermostability of CRISPR–Cas12b using Molecular Dynamics Simulations

Yinhao Jia[1], Katelynn Horvath[2], Santosh R. Rananaware[1], Piyush K. Jain[1,3,4], Janani Sampath[1,*]

[1]Department of Chemical Engineering, University of Florida, Gainesville, FL, USA

[2] Department of Chemical and Biomolecular Engineering, University of Connecticut, Storrs, CT, USA

[3] Department of Molecular Genetics and Microbiology, College of Medicine, University of Florida, Gainesville, FL, USA

[4] Health Cancer Center, University of Florida, Gainesville, FL, USA

* jsampath@ufl.edu

Table of content

Figure S1 BrCas12b domain assignment with detailed legend

Figure S2 All to all RMSD plot for the assessment of sampling during the first 200ns equilibration simulation

Figure S3 Total number of Intra protein hydrogen bond and total solvent accessible surface area of WT and MT at 300 K and 400 K

Figure S4 Secondary structure analysis of WT and MT at 300 K and 400 K

Figure S5 Predicted circular dichroism spectra using PDBMD2CD server

Figure S6 Backbone root mean squared deviation (RMSD) of each domain from WT and MT at 300 K and 400 K

Figure S7 Number of hydrogen bonds formed between the mutated residues and other residues of WT and MT at 300 K and 400 K

Figure S8 Protein dynamics projected onto PC2 from WT and MT at 300 K and 400 K

Figure S9 Projections of the simulation trajectory onto first two principal components

Figure S10 Cumulative contribution of all the principal components

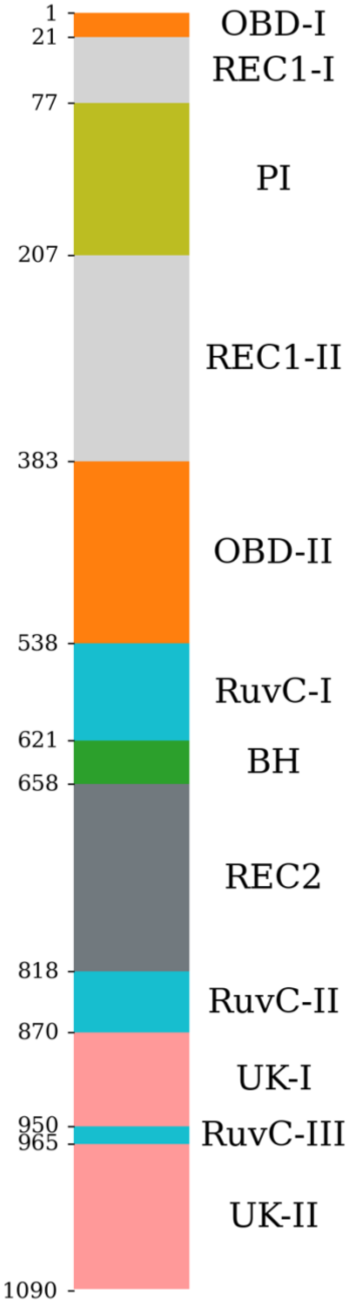

Figure S1. BrCas12b domain assignment.

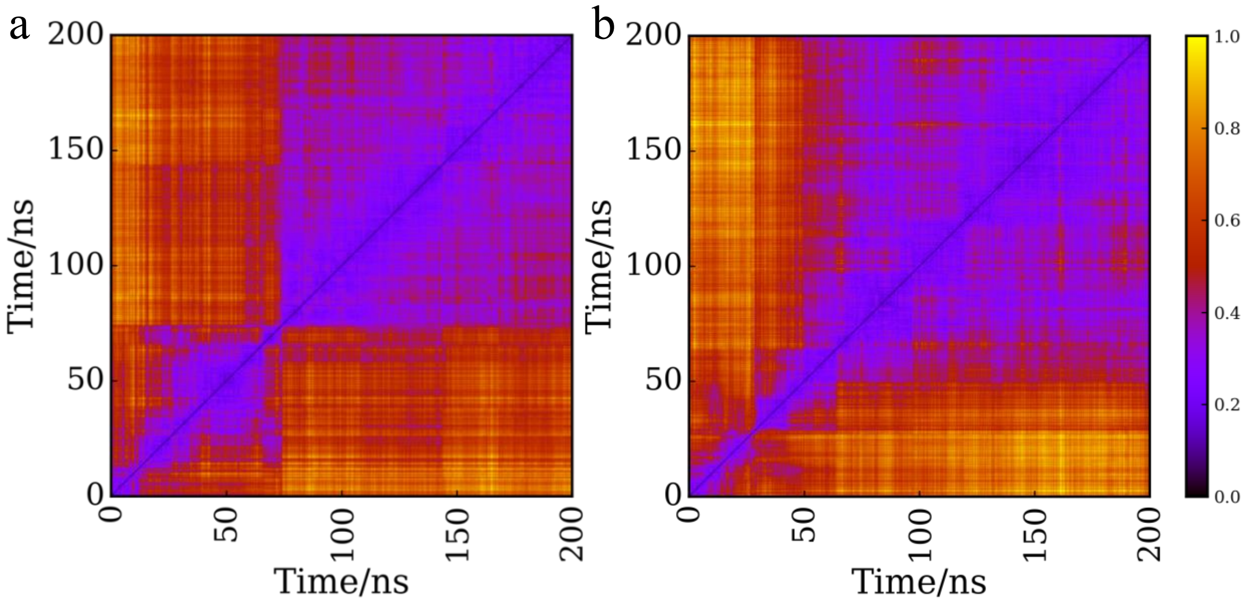

Figure S2. All to all RMSD of (a) wild-type BrCas12b and (b) mutated-type BrCas12b for the 200ns equilibration before random picking of configuration for the final simulation.

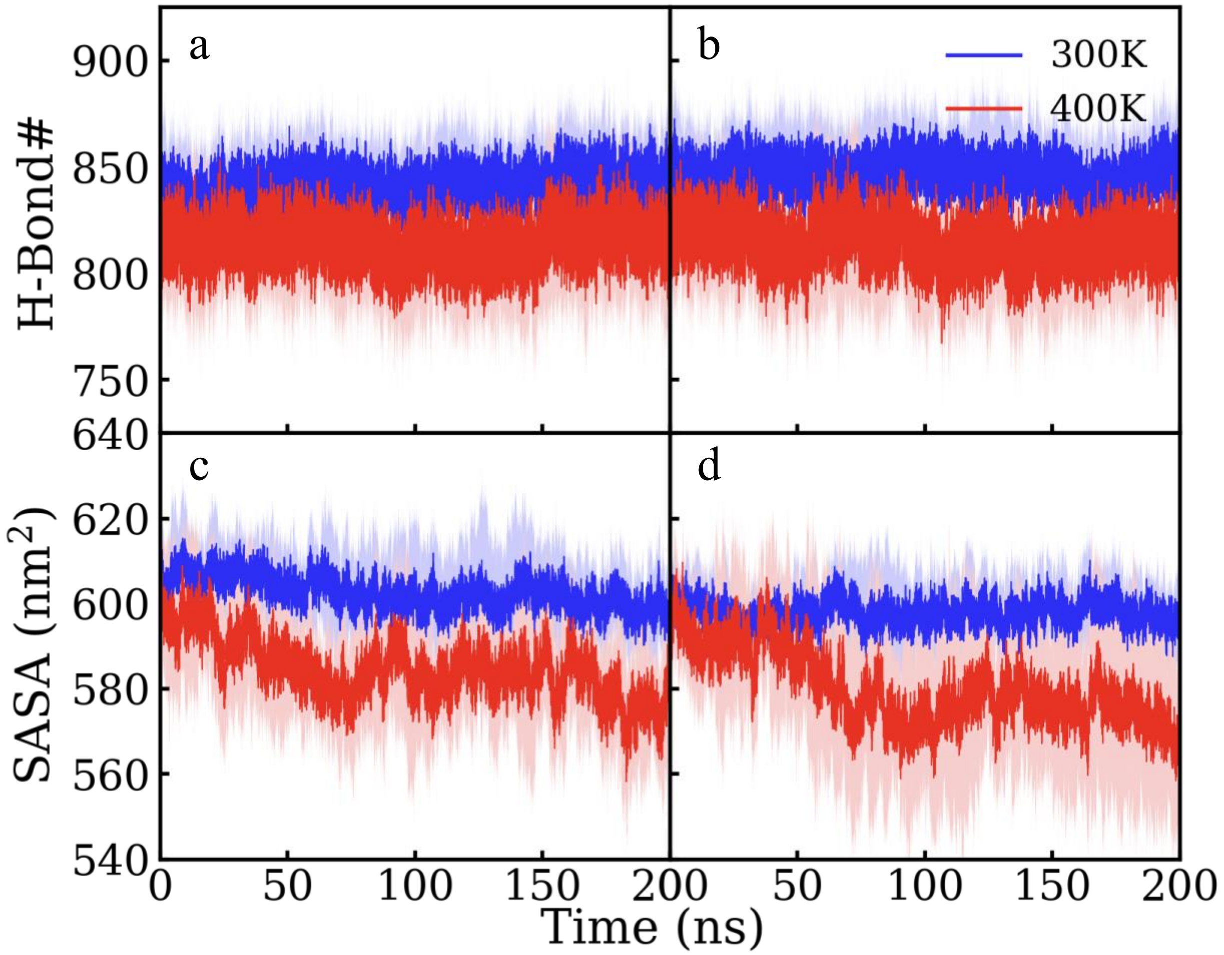

Figure S3. Intra protein hydrogen bond number (a, b) and total solvent accessible surface area (SASA) (c, d) of BrCas12b (a, c) wild type and (b, d) mutant type as a function of simulation time. The solid lines indicate values averaged over three independent trails, while the shaded region indicates the error calculated from the four replicas.

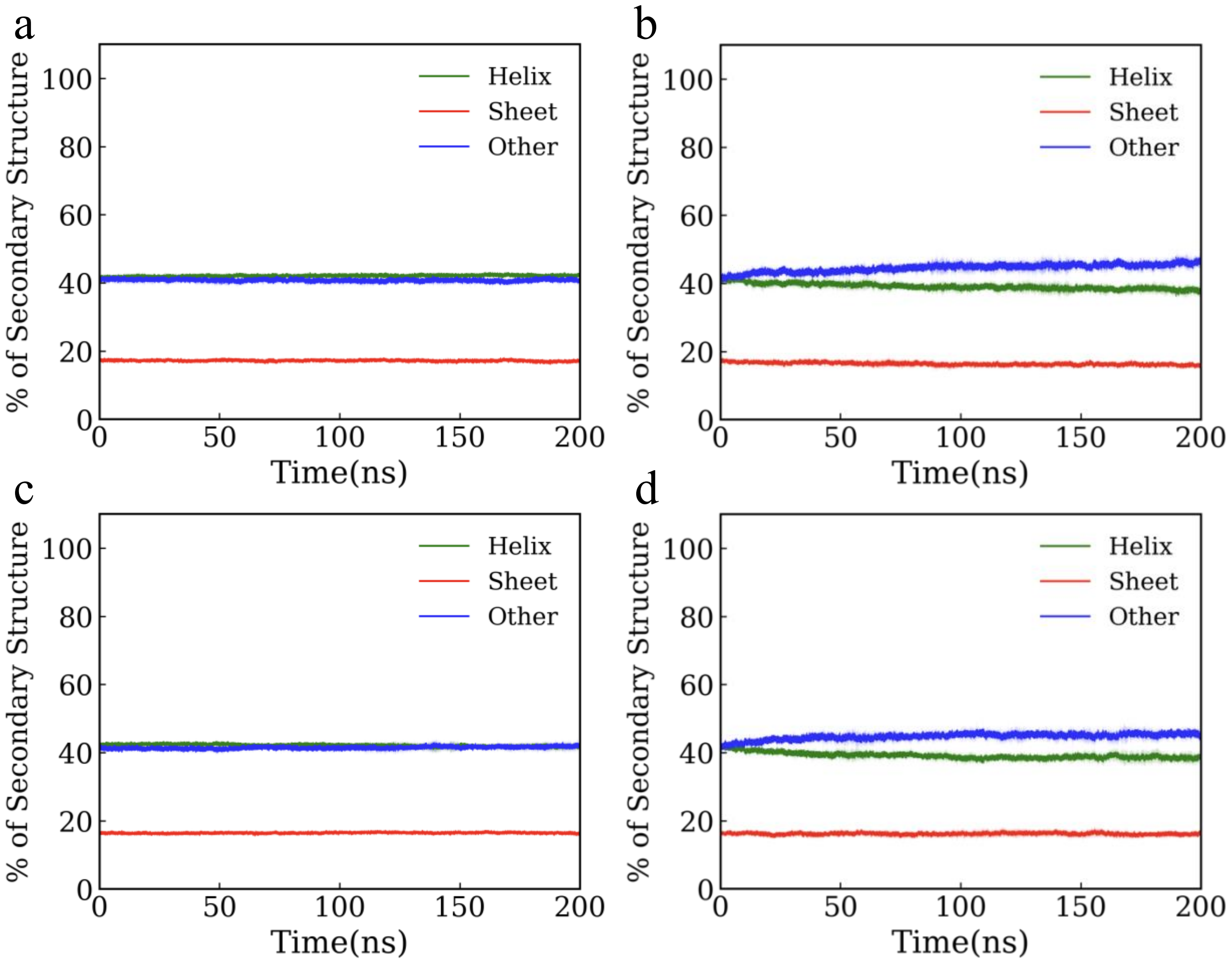

Figure S4. Secondary structure fraction of wild-type BrCas12b at (a) 300 K, (b) 400 K, and mutated-type BrCas12b at (c) 300 K, (d) 400 K. The solid lines indicate values averaged over three independent trails, while the shaded region indicates the error bar calculated from the three replicas.

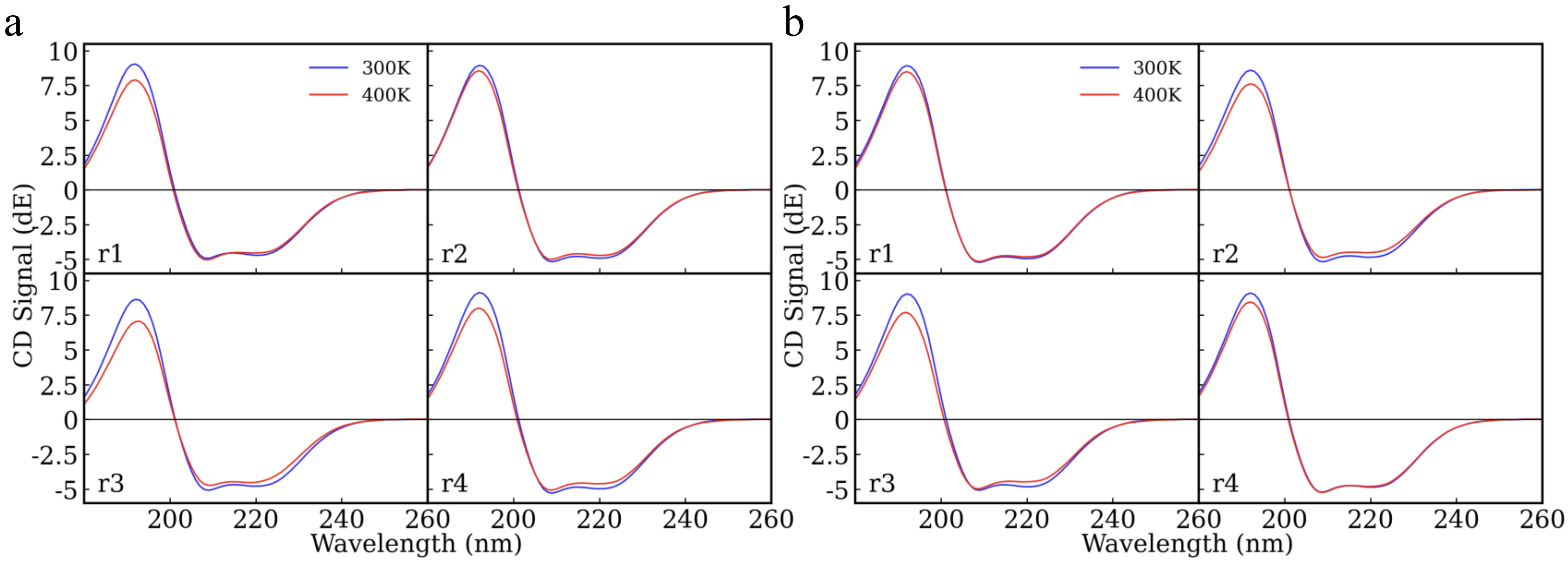

Figure S5. Predicted circular dichroism (CD) spectra of (a) wild-type BrCas12b and (b) mutated-type BrCas12b at 300 and 400 K after 200 ns simulation for four replicates (r1 – r4).

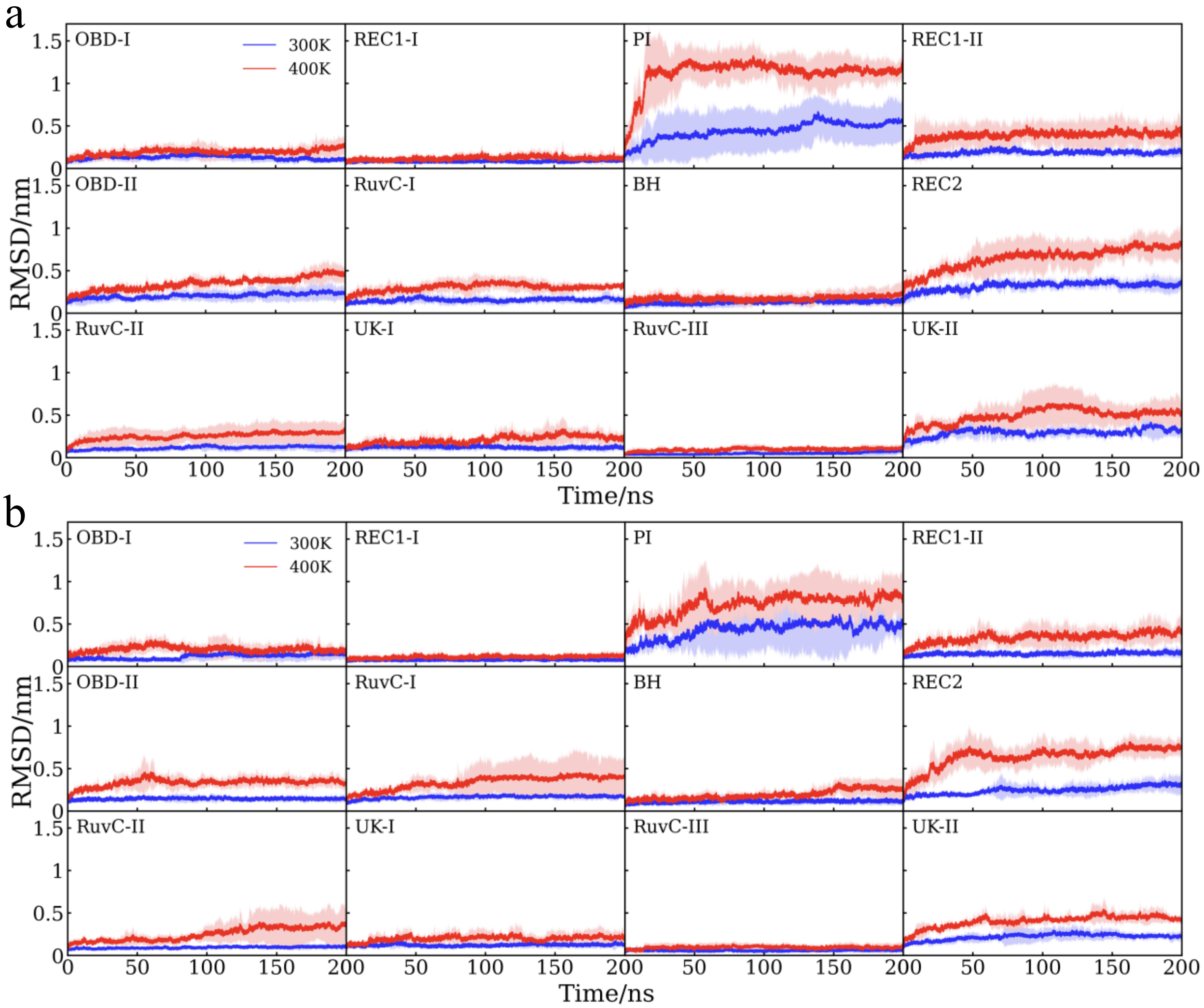

Figure S6. Backbone root mean squared deviation (RMSD) of each domain from (a) wild-type BrCas12b and (b) mutated-type BrCas12b, with the names of the domains shown at the top left corner. The solid lines indicate values averaged over three independent simulations, while the shaded region indicates the error bar calculated from the three replicas.

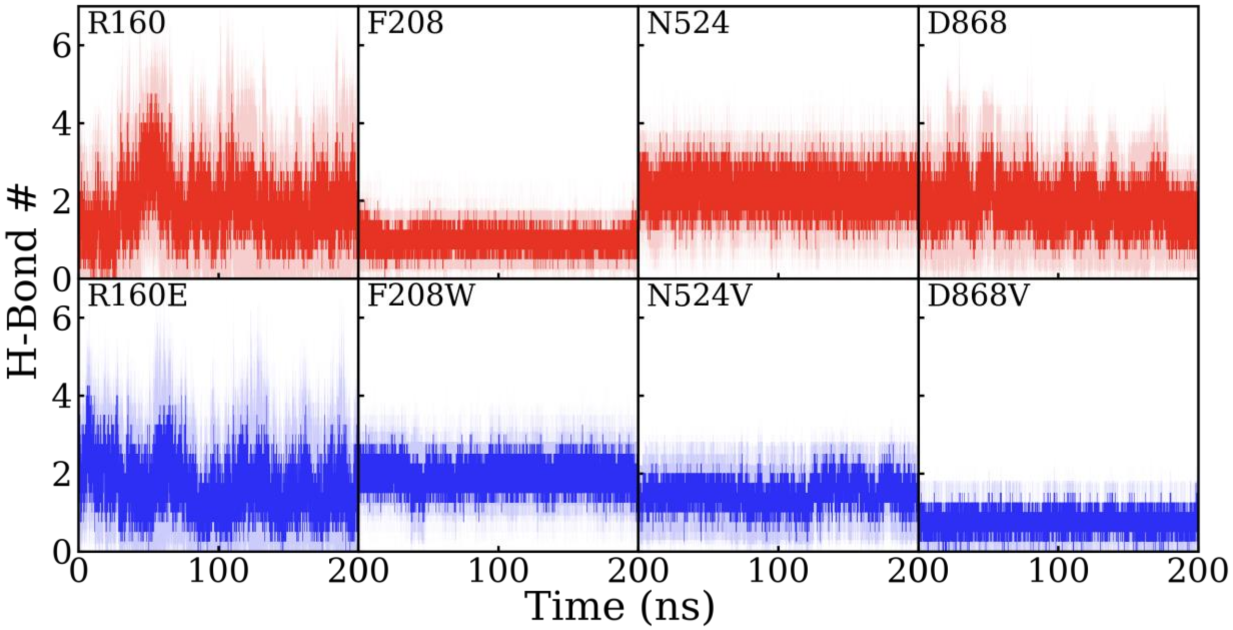

Figure S7. Number of hydrogen bond formed between the mutation point to other residues at 400 K, with each mutation point shown on the top left corner, as a function of simulation time. First row from wild-type, second row from mutated-type BrCas12b. The solid lines indicate values averaged over three independent trails, while the shaded region indicates the error bar calculated from the four replicas.

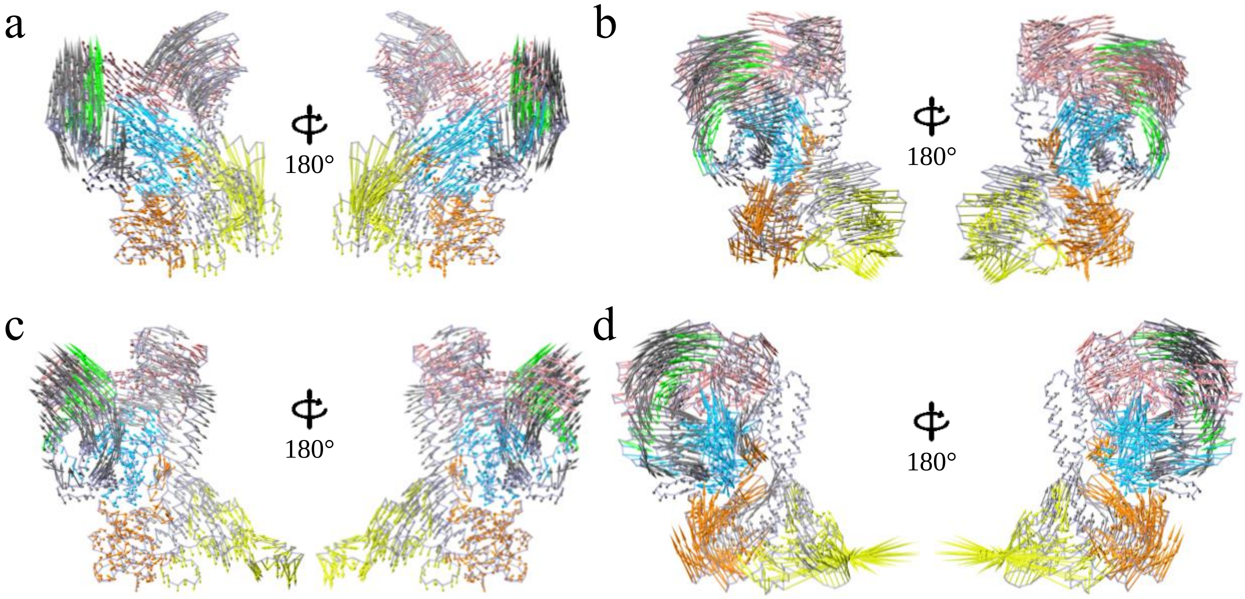

Figure S8. Motions obtained from principal component 2 (PC2) of wild-type BrCas12b at (a) 300 K, (b) 400 K, and mutated-type BrCas12b at (c) 300 K, (d) 400 K shown using arrows of sizes equivalent to the amplitude of motions, with colors adapted from Figure 1 to distinguish motions from different domains.

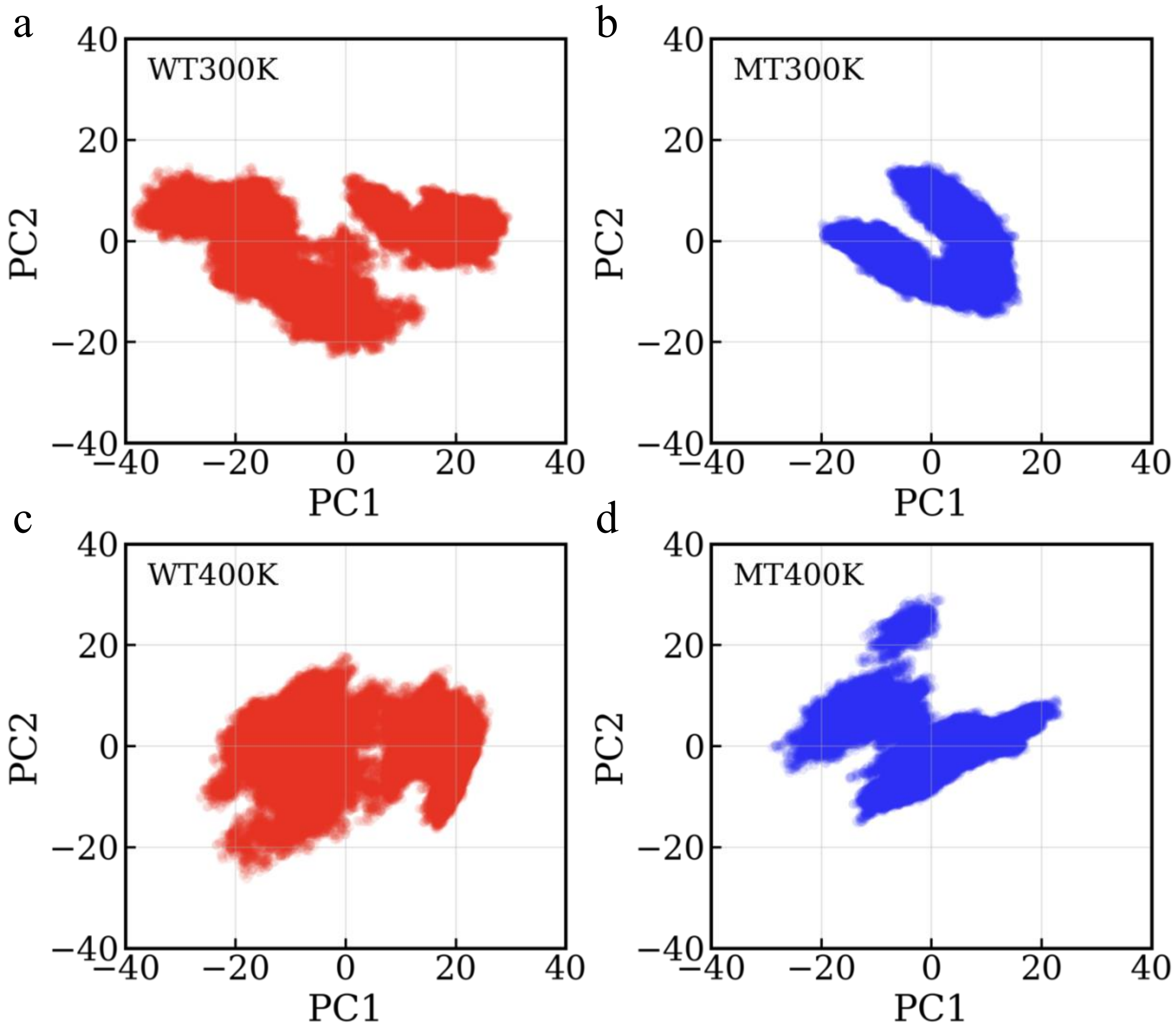

Figure S9. Projections of the simulation trajectory onto first and second principal component derived from (a) wild type BrCas12b at 300 K, (b) mutated type BrCas12b at 300 K, (c) wild type BrCas12b at 400 K, and (d) mutated type BrCas12b at 400 K.

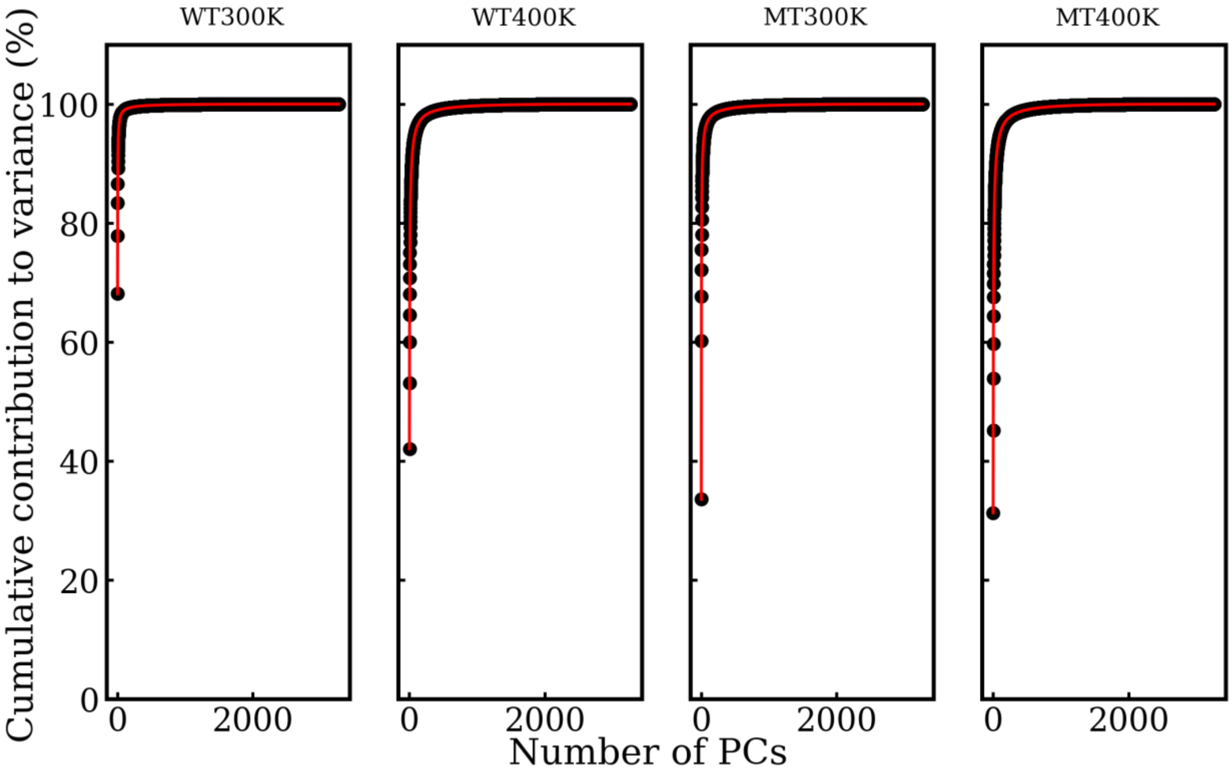

Figure S10. Cumulative contribution (%, y-axis) of all the principal components (PCs, x-axis) to the variance of the overall Cas12b motions calculated upon Principal Component Analysis (PCA) of WT and MT BrCas12b at 300 K and 400 K.
